# Development of self-stigma scale for adolescents in conflict with the law and its association with implicit self-stigma

**DOI:** 10.3389/fpsyg.2026.1767941

**Published:** 2026-09-21

**Authors:** Xiaoyi Wang, Zijiao Zhao, Jianglin Fu, Bangman Cai, Weijie Meng

**Affiliations:** 1 College of Education, Ludong University, Yantai, China; 2 Institute for Education and Treatment of Problematic Youth, Ludong University, Yantai, China; 3 Guiyang No.45 Middle School, Guiyang, China; 4 Sanbaiti Middle School, Renhuai, China

**Keywords:** self-stigma, justice-involved adolescents, implicit relational assessment procedure (IRAP), DAARRE model, conditional dissociation

## Abstract

**Introduction:**

Research on self-stigma among justice-involved adolescents is limited by a lack of dedicated instruments and an overreliance on explicit self-reports susceptible to social desirability bias.

**Methods:**

Two studies were conducted. Study 1 developed and validated the Self-Stigma Scale for Adolescents in Conflict with the Law (SSS-ACL), confirming a two-factor structure comprising perceived and internalized stigma with sound psychometric properties. Study 2 employed two IRAP protocols based on the DAARRE model to capture spontaneous relational responding for each dimension.

**Results:**

This showed that 81.08% of participants displayed positive implicit perceived stigma (automatic group-negative associations), whereas only 25.68% displayed positive implicit internalized stigma (automatic negative self-linkages); he two implicit indices were uncorrelated. Explicit-implicit correlations were conditional: explicit internalized stigma was unrelated to both IRAP indices, explicit perceived stigma was only marginally associated with its implicit counterpart, and explicit perceived stigma showed a small positive correlation with implicit internalized stigma.

**Discussion:**

These findings suggest that perceived and internalized stigma are functionally distinct at both reflective and spontaneous levels, and that robust implicit perceived stigma does not automatically translate into internalized self-stigma, consistent with a possible self-protective function. Practically, combining explicit and implicit measures may improve stigma assessment, and interventions should prioritize blocking internalization over merely reducing awareness of public stigma.

## Introduction

Stigma is a dynamic social process rooted in power relations, encompassing labeling, stereotyping, separation, status loss, and discrimination (Link and Phelan, 2001). Individuals who are labeled as devalued may internalize public prejudice into their self-concept, a phenomenon termed self-stigma. Corrigan and Kleinlein (2005) conceptualized this internalization as a progressive pathway from perceived stigma (awareness of public stereotypes) to internalized stigma (acceptance of those stereotypes as self-descriptive). Although this perception-internalization framework offers a foundational account of self-stigma, its applicability to adolescents in conflict with the law remains empirically untested and conceptually underdeveloped.

### The dual roots of self-stigma in justice-involved adolescents

Justice-involved adolescents are particularly vulnerable to self-stigma due to both social and developmental factors. Socially, the stigma they face is uniquely severe because of its moralized nature: unlike stigmas linked to uncontrollable conditions such as mental illness, illegal behavior is typically attributed to personal, controllable moral failure. This internal attribution lends perceived legitimacy to public prejudice, eliciting stronger moral outrage, greater social distance, and more punitive reactions (Moore et al., 2018; Pachankis et al., 2018; Côté-Lussier, 2016). When these youth are framed as inherently violent “superpredators” rather than as “wayward youth” who have temporarily lost direction, public support shifts from rehabilitation toward harsh punishment (Katzman et al., 2022; Greene et al., 2017). Consequently, they perceive exceptionally high levels of devaluation, which in turn amplifies the risk that public stigma will be internalized.

From a developmental perspective, adolescence is a period of heightened sensitivity to social evaluation, making it a critical window for stigma internalization (Earnshaw et al., 2022). Justice-involved youth are actively forming their identities, and negative labels imposed by the legal system are readily absorbed into their self-concepts. Formal legal processing typically induces stigma consciousness and endorsement of criminal stereotypes (Moore et al., 2016). This internalizing tendency enables perceived devaluation to translate directly into self-identity—the core of self-stigma.

The confluence of harsh external stigma and internal susceptibility produces complex psychosocial outcomes, including low self-esteem, depression, anxiety, and social withdrawal (Jolliffe et al., 2019; Baker et al., 2023). Efforts to conceal a stigmatized identity may further deepen internalization (Le Forestier et al., 2022). At the same time, a “what-the-hell” mentality can emerge: when individuals feel irrevocably branded as “bad,” they may experience cognitive dissonance and moral disengagement, leading them to embrace the delinquent label and engage in further deviance as a distorted means of seeking belonging and identity confirmation (Kamans et al., 2009). Such reactions widen the chasm between these adolescents and mainstream society, elevate recidivism risk, and pose a serious obstacle to successful reintegration. Thus, accurately capturing the structure and depth of self-stigma in this population is a prerequisite to designing effective rehabilitative strategies.

### Adaptation and reconstruction of self-stigma dimensions for justice-involved adolescents

Existing theoretical models and measurement tools for self-stigma are poorly suited to justice-involved adolescents. Moore et al. (2016) adapted Corrigan’s mental-illness framework (Corrigan et al., 2003; Corrigan and Kleinlein, 2005) and validated a four-stage model among adult offenders—perceived stigma, stereotype agreement, internalized stigma, and anticipated stigma—capturing the sequential process of crime-related stigma. However, three limitations undermine its applicability to adolescents. First, the instrument was derived from a mental-illness scale through simple label substitution, ignoring the distinct moral origins of offending stigma and compromising content validity. Second, the scale was developed without input from the target population, undermining construct validity and reinforcing researcher–participant power asymmetries (Feingold, 2021). Third, the model has not been cross-culturally validated with adolescent samples, leaving its stability in developmental populations unknown. Thus, a dedicated, validated instrument for youth self-stigma remains lacking.

Given that adolescence is a critical period for identity formation, stigma experiences in this group likely differ from those of adults, warranting theoretical adaptation. Retaining the core processual logic of Moore et al.’s model, we propose a revised framework. Among institutionalized youth, anticipated stigma can be conceptually decomposed into a temporal extension of perceived stigma (fear of future public devaluation) and an intensifier of present internalized stigma (fear of future self-perception); integrating it into these two dimensions preserves conceptual coherence and reduces operational complexity. Stereotype agreement, which serves as a transitional mechanism rather than an independent component, empirically overlaps with perceived stigma in adolescent samples (Li et al., 2010), and our pilot data indicated that agreement and internalization items were largely indistinguishable in correctional settings, likely because group and personal identities were fused. Accordingly, its content is distributed across the perceived and internalized dimensions to minimize redundancy and collinearity.

Based on these adaptations, we conceptualize youth self-stigma along two core dimensions: perceived stigma (awareness of public devaluation) and internalized stigma (incorporation of negative evaluations into the self-concept). Each dimension is operationalized through cognitive, affective, and behavioral facets. This developmentally grounded and pragmatically streamlined framework yields a parsimonious, theoretically sound model and a corresponding standardized scale, providing a reliable tool for future research and intervention.

### Implicit measurement: Why the IRAP?

Explicit self-report measures are inherently limited by social desirability and defensive self-presentation, a concern particularly acute among justice-involved adolescents in custodial settings, where surveillance and evaluative pressure amplify impression management and risk distorting self-stigma estimates (Blyth et al., 2023). Complementary approaches that bypass controlled self-presentation are therefore needed.

The present research adopts a Relational Frame Theory (RFT; Hayes et al., 2001) framework, conceptualizing IRAP response patterns as functional relational responses rather than latent mental entities (Brownstein et al., 2019; Barnes-Holmes and Harte, 2022). Self-stigma is thus viewed as relational network outputs, not fixed cognitive structures. Recent RFT advances—particularly the DAARRE model (Finn et al., 2018, 2019)—suggest that the IRAP can disentangle semantic (Crel) from emotional/attentional (Cfunc) properties, a distinction critical for capturing the shame and fear embedded in self-stigmatizing beliefs among justice-involved adolescents.

To capture these automatic responses, we employed the Implicit Relational Assessment Procedure (IRAP), an RFT-based measure assessing specific stimulus relations (Barnes-Holmes et al., 2006). The IRAP offers three advantages over the IAT. First, its design is prospectively grounded in RFT, whereas the IAT’s formal models were developed post hoc, creating conceptual ambiguity (Brownstein et al., 2019; Corneille and Hütter, 2020). Second, the IRAP shows greater resistance to faking (Qian, 2017) and targets overlearned relational patterns, which are less vulnerable to transient fluctuations. Third, the IRAP yields four independent D-scores (versus the IAT’s single relative score), allowing separate indexing of implicit perceived stigma and implicit internalized stigma, directly paralleling our explicit two-dimensional framework. By accessing overlearned relational components, the IRAP may reveal dissociations from explicit self-report that carry unique predictive value. However, no study has yet combined explicit and implicit self-stigma measures within this RFT framework in justice-involved adolescents—a gap the present study was designed to fill.

### The present studies

The present research comprises two studies. Study 1 developed and validated the Self-Stigma Scale for Adolescents in Conflict with the Law (SSS-ACL), establishing a developmentally appropriate two-dimensional structure comprising perceived stigma and internalized stigma. Study 2 employed the IRAP to measure the implicit counterparts of these dimensions and examined their convergence with explicit indices. Together, the two approaches provide a comprehensive account of self-stigma and inform targeted interventions.

## Study 1

### Materials and methods

#### Ethics Statement

This study received approval from the Institutional Review Board of Ludong University (Approval No. LDU-IRB2026041003) on April 10, 2026. Informed consent was obtained from all participants prior to data collection.

#### Participants

Four samples were collected via convenience sampling across multiple phases, each serving distinct analytical purposes (Table 1).

**TABLE 1:** Overview of sample.

Sample	Total distributed	Valid responses	Gender (*N*)	*M*_age_ ± *SD*	Purpose
Sample1	772	720	*N*_male_ = 602 *N*_female_ = 118	14.60 ± 1.98	Item analysis and exploratory factor analysis (EFA)
Sample 2	480	388	*N*_male_ = 339 *N*_female_ = 49	14.78 ± 1.14	Confirmatory factor analysis (CFA) and criterion validity
Sample 3	331	266	*N*_male_ = 222 *N*_female_ = 44	15.00 ± 1.17	Test-retest reliability analysis
Sample 4	–	772	*N*_male_ = 664 *N*_female_ = 108	14.68 ± 1.19	Measurement invariance testing

Sample 4 was drawn from the combined participant pool across the preceding phases, restricted to those with complete and accurate self-reports of offense history. Offense type distribution in Sample 4: first-time offenders (*n* = 643), recidivists (*n* = 129); property-related (*n* = 438), personal injury-related (*n* = 142), mixed offenses (*n* = 192).

#### Procedure and measures

Following institutional approval, researchers coordinated with correctional education staff to finalize the data collection schedule. On testing days, participants received informed consent forms and confidentiality agreements. Those who agreed provided written consent; those who declined were excused without penalty. With the exception of adolescents with extremely limited literacy, all participants completed the preliminary scale under researcher guidance.

##### Preliminary self-stigma scale for adolescents in conflict with the law (SSS-ACL)

The initial item pool was generated through a multi-step process. First, a systematic literature review of self-stigma measures used with marginalized populations (e.g., adult offenders, individuals with mental illness, substance users) informed item adaptation. Second, in-depth interviews and open-ended surveys with justice-involved adolescents were conducted to capture lived experiences. Third, a panel of four psychology experts (professors and doctoral students) evaluated item relevance, clarity, and cultural appropriateness. Experts rated each item on a 4-point relevance scale (1 = *not relevant*, 4 = *highly relevant*); items with Item-Level Content Validity Indices (I-CVIs) ≥ 0.75 were retained, and written feedback guided wording refinements. Finally, a pilot test with 40 randomly selected adolescents from a correctional school in S Province assessed readability and comprehension. These procedures yielded a preliminary 44-item version of the SSS-ACL.

##### Core Self-Evaluations Scale (CSES)

The revised Chinese version of the Core Self-Evaluations Scale (Du et al., 2012) was administered. The scale comprises 10 items rated on a 5-point Likert scale (1 = *strongly disagree*, 5 = *strongly agree*), with Items 2, 3, 5, 7, 8, and 10 reverse-scored. In the current study, Cronbach’s α = 0.787.

##### Rosenberg Self-Esteem Scale (RSES)

The Chinese adaptation of the Rosenberg Self-Esteem Scale (Yan et al., 2021) was used. It consists of 10 items rated on a 4-point scale (1 = *strongly agree*, 4 = *strongly disagree*), with higher total scores indicating greater self-esteem. Cronbach’s α = 0.736.

##### Perceived discrimination questionnaire

The Perceived Discrimination Questionnaire (Shen et al., 2009) was employed. This 6-item measure comprises two subscales (personal and group discrimination, 3 items each) rated on a 5-point Likert scale. The mean score indexes perceived discrimination, with higher scores reflecting greater perceived discrimination. Cronbach’s α = 0.839.

#### Statistical analysis

Item analysis (critical ratio, corrected item-total correlation, and homogeneity tests) was performed using SPSS 27.0, retaining items meeting satisfactory criteria (Wu, 2010). Multiple exploratory factor analyses (EFAs) with factor rotations were then conducted in R (Version 4.6.0; R Core Team, 2025) using the psych (Revelle, 2021) and GPArotation (Bernaards and Jennrich, 2005) packages to derive a stable factor structure. Multi-group confirmatory factor analysis (CFA) was subsequently carried out using the lavaan (Rosseel, 2012) and semTools (Jorgensen et al., 2012) packages to test measurement invariance across offense frequency and offense type.

### Results

#### Item analysis

Item analysis of the initial 44 items was conducted using the critical ratio (CR), corrected item–total correlations, and homogeneity tests. Items were removed if they met any of the following criteria: (a) nonsignificant CR or CR < 3.00 (Wu, 2010); (b) nonsignificant corrected item–total correlation or *r* < 0.30 (Nunnally and Bernstein, 1994); (c) deletion would increase the overall *Cronbach’s α* above 0.882, or communality < 0.20 and factor loading < 0.45. A total of 21 items were eliminated, leaving 23 items for exploratory factor analysis. Detailed statistics are provided in Supplementary Table S1.

#### Exploratory factor analysis

We first examined the distributional properties of the 23 retained items. Skewness ranged from -0.31 to 1.05 and kurtosis from -0.92 to 0.39, indicating notable deviations from normality for several items. A polychoric correlation matrix was therefore used to estimate item associations, avoiding potential bias from Pearson correlations with ordinal data. The Kaiser-Meyer-Olkin (KMO) measure was 0.86, and Bartlett’s test of sphericity was significant, χ^2^(253) = 5476.32, *p* < 0.001. Based on the screen plot (Figure 1), the MAP test (minimum at 2 factors, MAP = 0.013), and the theoretical two-dimensional model, two factors were extracted using maximum likelihood estimation with oblimin rotation.

**FIGURE 1:**
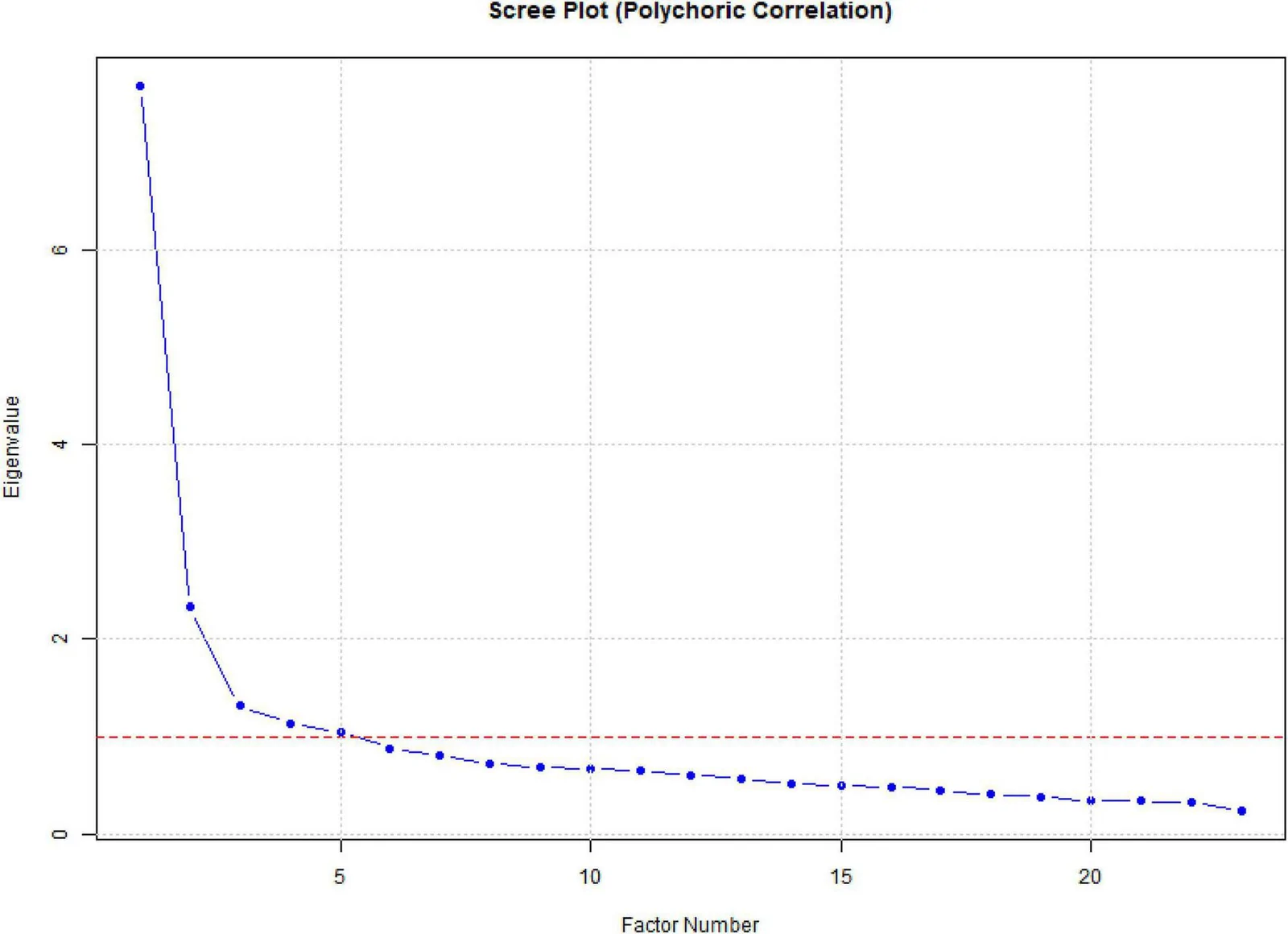
Scree plot.

An iterative EFA was then conducted on the 23 items. At each iteration, items were removed if they met any of the following criteria: (a) primary factor loading <0.50; (b) absolute difference between the primary and the next highest loading < 0.20 (with primary loading >0.30); or (c) communality < 0.20. After eight iterations, seven items (s1, s5, s16, s23, s25, s26, s40) were dropped, yielding a 16-item scale. Factor loadings and communalities are shown in Table 2. Factor 1 (Perceived Stigma) comprised seven items with loadings ranging from 0.586 to 0.808, capturing awareness of public stigma. Factor 2 (Internalized Stigma) comprised nine items with loadings ranging from 0.541 to 0.851, reflecting the incorporation of public devaluation into the self-concept. The two factors were moderately correlated (*r* = 0.53) and together accounted for 45.17% of the total variance. Communalities ranged from 0.316 to 0.658. Model fit was acceptable: *RMSEA* = 0.075 (90% CI [0.068, 0.082]), *CFI* = = 0.923, *TLI* = 0.895, *SRMR* = 0.041. Although the χ^2^ test was significant, χ^2^(89) = 445.63, *p* < 0.001, this was expected given the large sample size.

**TABLE 2:** Factor loadings and communalities for the final 16-item two-factor EFA model (*N* = 720).

Items	Rotated factor loadings		Communality
	Perceived (F1)	Internalized (F2)	
s7 Most people are unwilling to work with someone who was once in trouble with the law, like me	0.81		0.65
s8 Most people feel disgusted with young people like me who have been in trouble with the law	0.75		0.56
s11 Most people look down on young people like me who have been in trouble with the law	0.72		0.52
s6 Most people think that young people like me who have been in trouble with the law are dangerous	0.71		0.56
s12 Most people do not value the suggestions or opinions of young people like me who have been in trouble with the law	0.65		0.35
s10 Most people think young people like me who have been in trouble with the law are untrustworthy and unreliable	0.62		0.45
s13 Most people think young people like me who have been in trouble with the law are incapable of repenting	0.59		0.36
s31 I feel inferior because of my offense history		0.85	0.66
s28 I hate myself because of my past offenses		0.74	0.52
s36 I feel that I will become lonelier in the future because of social exclusion		0.65	0.44
s33 I feel that my past offenses have cost me my social standing		0.60	0.39
s30 I feel that I am a danger to society or to others		0.55	0.40
s18 I worry or fear that others will find out about my offense history and experiences		0.55	0.38
s21 Because of my offense history, I avoid contacting former classmates or relatives		0.55	0.32
s34 I expect that I will try my best to hide my offense history and identity		0.54	0.32
s29 Even if I sincerely repent, people will not truly trust me		0.54	0.35
Eigenvalue	3.44	3.58	
Cumulative variance explained (%)	22.15	45.17	

*N* = 720. Factor loadings < 0.30 are omitted. Perceived = perceived stigma; Internalized = internalized stigma.

#### Confirmatory factor analysis

To cross-validate the EFA-derived structure, a confirmatory factor analysis (CFA) using WLSMV estimation was performed on an independent sample (*n* = 388). The two-factor model demonstrated good fit to the data (Table 3): χ^2^(103) = 237.52, *p* < 0.001, χ^2^/df = 2.31, RMSEA = 0.058 (90%CI [0.048, 0.068]), CFI = 0.993, TLI = 0.992, SRMR = 0.047. A competing one-factor model demonstrated significantly worse fit, *Δχ*^2^(1) = 139.87, *p* < 0.001) supporting the two-dimensional structure.

**TABLE 3:** Model fit indices for confirmatory factor analysis (*N* = 388).

Model	χ^2^	*df*	χ^2^/*df*	*RMSEA [90% CI]*	*SRMR*	*CFI*	*TLI*
One-factor model	855.83	104	8.23	0.137 [0.128, 0.145]	0.090	0.961	0.955
Two-factor model	237.52	103	2.31	0.058 [0.048, 0.068]	0.047	0.993	0.992

*N = 388.* RMSEA, root mean square error of approximation; SRMR, standardized root mean square residual; CFI, comparative fit index; TLI, Tucker-Lewis index.

#### Validity

##### Construct validity

Standardized factor loadings, composite reliability (CR), and average variance extracted (AVE) are reported in Table 4. All loadings were significant (*p* < .001) and ranged from 0.560 to 0.824. CR values were 0.896 for perceived stigma and 0.890 for internalized stigma, exceeding the recommended threshold of 0.70. AVE values were 0.554 for perceived stigma and 0.476 for internalized stigma, indicating acceptable convergent validity. The square roots of AVE (0.745 and 0.690) exceeded the inter-factor correlation (*r* = 0.678), supporting discriminant validity. Harman’s single-factor test further supported the distinctiveness of the two factors: the one-factor model fit substantially worse (CFI = 0.961, RMSEA = 0.137) than the two-factor model.

**TABLE 4:** Standardized factor loadings, composite reliability, and average variance extracted.

Latent variable	Item	Standardized loading	*CR*	*AVE*
Perceived stigma			0.896	0.554
	s8	0.824		
	s7	0.794		
	s6	0.787		
	s11	0.787		
	s10	0.717		
	s13	0.676		
	s12	0.601		
Internalized stigma			0.890	0.476
	s31	0.780		
	s30	0.762		
	s36	0.745		
	s28	0.710		
	s18	0.696		
	s33	0.678		
	s29	0.627		
	s34	0.625		
	s21	0.560		

##### Criterion-related validity

As displayed in Table 5, SSS-ACL total and subscale scores correlated significantly with core self-evaluation (*rs* = –0.52 to –0.37), self-esteem (*rs* = –0.50 to –0.30), and perceived discrimination (*rs* = = 0.43 to 0.57), all in the expected directions. Internalized Stigma consistently showed stronger correlations with these criteria than did Perceived Stigma.

**TABLE 5:** Correlations among SSS-ACL scores and criterion variables.

Variable	*M*	*SD*	Perceived	Internalized	Total
1. Perceived stigma	3.44	1.06	–		
2. Internalized stigma	3.03	1.02	0.56***	–	
3. Total self-stigma	3.25	0.92	0.90***	0.86***	
4. Core self-evaluation	3.21	0.58	–0.37***	–0.56***	–0.52***
5. Personal discrimination	2.76	0.90	0.28***	0.50***	0.43***
6. Group discrimination	2.93	0.99	0.40***	0.54***	0.52***
7. Total discrimination	2.85	0.87	0.38***	0.57***	0.53***
8. Self-esteem	2.79	0.45	–0.30***	–0.50***	–0.44***

*N* = 388. Perceived = perceived stigma; Internalized = internalized stigma; Total = total self-stigma.

*** *p* < 0.001.

#### Reliability

Internal consistency and test-retest reliability are reported in Table 6. Cronbach’s α was 0.90 for the total scale, 0.87 for the Perceived Stigma subscale, and.85 for the Internalized Stigma subscale. Two-week test–retest correlations ranged from 0.74 to 0.79, with all lower 95% CI bounds exceeding 0.65, indicating good internal consistency and temporal stability.

**TABLE 6:** Reliability estimates.

Factor	Cronbach’s α	Test–retest r[95% CI]
Perceived stigma	0.87	0.74[0.68, 0.79]
Internalized stigma	0.85	0.75[0.69, 0.80]
Total scale	0.90	0.79[0.74, 0.83]

#### Measurement invariance

Measurement invariance was examined across offense frequency (first-time vs. repeat) and offense type (property, personal, mixed). As shown in Tables 7, 8, the configural invariance models fit well for both grouping variables, and changes in *CFI* and *RMSEA* from the configural to the strict invariance models were minimal (ΔCFI* ≤* 0.004 and ΔRMSEA ≤ 0.003). These results support full strict invariance, thereby permitting meaningful mean comparisons across subgroups.

**TABLE 7:** Measurement invariance across offense frequency.

Model	χ^2^	*df*	*TLI*	*CFI*	*RMSEA (90% CI)*	Δ*TLI*	Δ*CFI*	Δ*RMSEA*
M1 configural	392.051	172	0.914	0.929	0.059 [0.052, 0.065]			
M2 weak	406.45	185	0.919	0.929	0.057[0.050, 0.063]	0.005	0.000	–0.002
M3 strong	426.25	198	0.922	0.927	0.056[0.049, 0.062]	0.003	–0.002	–0.001
M4 strict	439.06	213	0.928	0.927	0.053[0.047, 0.060]	0.006	0.000	–0.003

M1 = baseline; M2 = equal loadings; M3 = equal loadings + intercepts; M4 = equal loadings + intercepts + residual variances. Δ = difference between adjacent models.

**TABLE 8:** Measurement invariance across offense type.

Model	χ^2^	*df*	*TLI*	*CFI*	*RMSEA (90% CI)*	Δ*TLI*	Δ*CFI*	Δ*RMSEA*
M1 configural	516.631	258	0.900	0.918	0.064 [0.057, 0.071]			
M2 weak	546.407	284	0.908	0.917	0.061 [0.054, 0.068]	0.008	–0.001	–0.003
M3 strong	584.826	310	0.912	0.913	0.060 [0.053, 0.066]	0.004	–0.004	–0.001
M4 strict	622.379	340	0.917	0.911	0.058 [0.051, 0.064]	0.005	–0.002	–0.002

Same as Table 9.

### Discussion

This study developed and validated the Self-Stigma Scale for Adolescents in Conflict with the Law (SSS-ACL) within a perceived-internalized two-dimensional framework and confirmed its factor structure in an independent sample. Results indicate that self-stigma among justice-involved adolescents can be parsimoniously represented by two related yet distinguishable dimensions: perceived stigma (awareness of public devaluation) and internalized stigma (incorporation of negative evaluations into the self-concept).

This two-dimensional structure aligns with Corrigan and Kleinlein’s (2005) distinction between public stigma and self-stigma from the target’s perspective. However, it diverges from the four-stage model (perceived stigma, stereotype agreement, internalized stigma, anticipated stigma) validated by Moore et al. (2016) with adult offenders. Two developmental features likely account for this divergence. First, the heightened salience of peer relationships and group identity during adolescence (Brown, 2004) may blur the boundary between public stereotypes and self-evaluation, such that perceived stigma is rapidly assimilated into the self-concept, bypassing the reflective stereotype-agreement stage observed in adults. Second, adolescents’ limited future time perspective (Nurmi, 1991) and ongoing identity formation may fuse anticipated discrimination with present perceptions of bias and self-devaluation, rather than maintaining it as an independent forward-looking construct (Fox et al., 2018; Eaton et al., 2020; McWilliams and Hunter, 2021). Thus, their stigma experiences reflect a concurrent interplay of past, present, and future pressures (Earnshaw et al., 2022). These findings underscore the necessity of developmental adaptation when applying adult-derived stigma models to younger populations.

Construct validity was supported by composite reliability values exceeding 0.89 for both factors and significant standardized loadings. Although the AVE for internalized stigma was 0.476, slightly below the 0.50 benchmark, this modest shortfall is acceptable given the adequate composite reliability and significant loadings (Fornell and Larcker, 1981), and likely reflects the multidimensional nature of internalized stigma, which encompasses self-devaluation, social withdrawal, and emotional distress. Discriminant validity was confirmed by the Fornell-Larcker criterion and the superior fit of the two-factor model over a single-factor model.

Criterion-related validity was demonstrated through theoretically expected correlations: SSS-ACL scores correlated negatively with self-esteem and core self-evaluation, and positively with perceived discrimination. Notably, internalized stigma consistently exhibited stronger associations with all criterion variables than perceived stigma, consistent with its role as a more proximal risk factor for psychological maladjustment (Corrigan et al., 2006; Quinn and Chaudoir, 2009). This pattern reinforces the view that interventions should prioritize blocking the internalization process rather than merely reducing awareness of public prejudice (Hatzenbuehler, 2016; Borschmann et al., 2020; Baker et al., 2023). Full strict measurement invariance was established across offense frequency and offense type, indicating that the two-dimensional structure is robust across subgroups and supporting the scale’s broad applicability.

Several limitations should be noted. First, the suboptimal AVE for internalized stigma suggests that future revisions could benefit from more homogeneous items targeting its core affective components. Second, gender and longitudinal invariance could not be examined due to sample constraints. Most importantly, the exclusive reliance on explicit self-report is vulnerable to social desirability, particularly in correctional settings. Study 2 therefore employed the IRAP to capture implicit perceived and internalized stigma, enabling a more comprehensive understanding of how self-stigma operates across controlled and automatic cognitive processes. Finally, because the SSS-ACL was developed in a Chinese collectivist context, cross-cultural validation in individualist societies is warranted to test the generalizability of the two-dimensional structure.

## Study 2

### Materials and methods

#### Participants

Given the time-intensive nature of the IRAP and the recommendation of at least 30 participants per group, we recruited a larger sample to ensure adequate power for explicit–implicit comparisons. After applying standard IRAP data-screening criteria (see Data Processing), the final matched sample comprised 74 adolescents (52 male, *M*_*age*_ = 14.91, *SD* = 1.06) with valid data on both IRAP tasks. All participants had normal or corrected-to-normal vision, at least junior high school education, no prior IRAP experience, and the ability to operate a computer independently. Participants received small gifts as compensation.

#### Materials

Based on the DAARRE model (Finn et al., 2018, 2019), trial-level effects in the IRAP depend on the synergy of relational (Crel) and emotional/attentional (Cfunc) stimulus functions. Combining perceived and internalized stigma within a single IRAP would cause their opposing emotional valences to dilute each other’s trial-level effects, obscuring the distinction between the two constructs. To avoid this mutual neutralization, we implemented two separate IRAP tasks—one for perceived stigma and one for internalized stigma—ensuring functional uniformity of the stimuli within each task.

Concept terms were selected through open-ended questionnaires and word evaluations in a pilot study. For the perceived-stigma IRAP, concept terms consisted of four labels describing justice-involved adolescents (“*youth who offended,” “young people with behavioral issues,” “justice-involved youth,” “young people who often get into trouble”*) four control labels (“*law-abiding youth,” “typical young people,” “average young people,” “well-behaved students”*). Attribute words comprised four positive terms (*“likeable,” “safe,” “competent*, *trustworthy”*) and four negative terms (*“avoided,” “impulsive,” “deceitful,” “dangerous”*). The same attribute words were used in the internalized-stigma IRAP, with concept words replaced by *“I am”* and *“I am not.”*

#### Procedure

The IRAP was programmed in PsychoPy (Peirce, 2007, 2009). On each trial, a concept word appeared at the top of the screen, an attribute word in the center, and response options (“Agree”/“Disagree”) at the bottom. Participants were instructed to respond as quickly and accurately as possible according to the current block rule. In consistent blocks, responses were congruent with common stereotypes (e.g., press “*Agree*” for justice-involved youth paired with negative attributes or law-abiding youth paired with positive attributes). In inconsistent blocks, the required responses were reversed.

The experiment comprised two separate IRAP tasks: a perceived-stigma IRAP and an internalized-stigma IRAP. Each IRAP task consisted of two practice blocks (one consistent, one inconsistent) followed by four test blocks (two consistent and two inconsistent, presented in alternating order). The order of the two IRAP tasks was counterbalanced across participants using a Latin-square design, and within each task, half of the participants began with a consistent block and half with an inconsistent block. The internalized-stigma IRAP followed the identical procedure except for the concept words. After completing both IRAPs, participants completed the SSS-ACL (Study 1) and a brief demographic questionnaire. The full block sequence is provided in Supplementary Table S3.

#### Data processing

IRAP D-scores adapt the IAT scoring logic (Greenwald et al., 2003) to the IRAP trial structure and were computed following Barnes-Holmes et al. (2010) and Hussey et al. (2015). Only test-block data were analyzed; practice blocks were excluded. Participants were removed if overall accuracy on test blocks fell below 80% or if more than 10% of test-block trials had latencies < 300 ms. Additionally, trials with latencies exceeding 10,000 ms were discarded.

For each IRAP task, the four formal test blocks were divided into two consecutive consistent–inconsistent block pairs (Blocks 3–4 and 5–6). For each pair, a D-score was computed using only correct-response latencies—because the IRAP paradigm requires correct responses to proceed—according to the formula: (mean inconsistent-block latency - mean consistent-block latency)/pooled standard deviation of the two blocks. The two pairwise D-scores were then averaged to yield a single task-specific D-score. Thus, the perceived-stigma and internalized-stigma IRAPs each produced one D-score (PS_D and IS_D, respectively). Positive D-scores indicate stronger automatic associations with negative attributes; negative D-scores indicate stronger associations with positive attributes. Consistent with contemporary revised frameworks (Greenwald and Banaji, 2017) and the Cfunc tenet of the DAARRE model, these D-scores capture brief, spontaneous relational responding under time pressure rather than stable unconscious dispositions. Subsequently, correlation analyses examined associations between implicit and explicit perceived stigma and between implicit and explicit internalized stigma to explore implicit-explicit dissociations.

### Results

#### Implicit self-stigma: overall pattern

The IRAP D-score for implicit perceived stigma was positive (*M* = 0.167, *SD* = 0.174), with 81.08% of participants showing a positive bias, indicating robust spontaneous relational responding linking the justice-involved youth label to negative attributes. In contrast, the D-score for implicit internalized stigma was negative (*M* = -0.116, *SD* = 0.171), with only 25.68% of participants displaying automatic negative self-associations. A paired-sample *t*-test revealed a significant difference between the two implicit dimensions, *t*(73) = 9.156, *p* < 0.001 (Table 9). This pattern suggests that, under time-pressured conditions, negative associations with the group label are readily activated even among these adolescents; however, for most, such group-level negativity does not spontaneously generalize to self-evaluation.

**TABLE 9:** Descriptive statistics for implicit stigma (*N* = 74).

Variable	*M*	*SD*	*n* (positive D)	% (positive D)
Implicit Perceived Stigma	0.167	0.174	60	81.08
Implicit Internalized Stigma	–0.116	0.171	19	25.68
*t*	9.156***			

***p < 0.001.

#### Relations between explicit and implicit stigma

Table 10 presents bivariate correlations among explicit and implicit indicators. Explicit perceived stigma was strongly positively correlated with explicit internalized stigma (*r* = 0.649, *p* < 0.001). Explicit perceived stigma showed a marginal positive correlation with implicit perceived stigma (*r* = 0.200, *p* = 0.09) and a small but significant positive correlation with implicit internalized stigma (*r* = 0.240, *p* < 0.05). Explicit internalized stigma was not significantly correlated with either implicit perceived stigma (*r* = 0.006, *p* > 0.05), or implicit internalized stigma (*r* = 0.107, *p* > 0.05). The two IRAP-derived D-scores were not significantly correlated (*r* = –0.185, *p* > 0.05).

**TABLE 10:** Correlations between explicit and implicit stigma (*N* = 74).

Variable	1	2	3	4
1 Explicit perceived	–	–	–	–
2 Explicit internalized	0.649***			
3 Implicit perceived	0.200^†^	0.006		
4 Implicit internalized	0.240*	0.107	–0.185	

† 0.05 *< p* < 0.10,

*p < 0.05,

***p < 0.001.

### Discussion

Study 2 yielded two central findings. First, implicit perceived stigma was robust and positive, whereas implicit internalized stigma was weak and negative, and the two IRAP-derived implicit indices were not significantly correlated. According to the DAARRE model and relational frame theory (Finn et al., 2018, 2019), in the perceived stigma task, group labels primed a strong negative context and automatically activated the relation “the group is devalued,” producing a positive D-score; in the internalized stigma task, self-reference activated a self-protective positive context that conflicted with negative group attributes, thereby impairing relational coherence and yielding a weak negative D-score. Justice-involved adolescents rapidly activate the group-devaluation frame because of widely available cultural representations of offending groups in their social environment, but they do not bind this frame to the self with equal strength, suggesting that protective mechanisms such as cognitive decoupling and defensive self-enhancement buffer the automatic transformation of external stigma into internalized stigma (Major and O’Brien, 2005; Corrigan and Rao, 2012).

Second, implicit-explicit associations were not uniform but rather conditional. Explicit internalized stigma was unrelated to both IRAP indices. Because it is highly self-relevant and thus subject to the strongest social desirability pressure, participants tend to avoid negative self-reports, consistent with meta-analytic evidence that social desirability attenuates implicit-explicit correlations (Hofmann et al., 2005). Explicit perceived stigma was only marginally correlated with implicit perceived stigma, likely because it does not directly implicate the self and therefore entails relatively lower self-presentational pressure. Notably, a small positive correlation emerged between explicit perceived stigma and implicit internalized stigma. Although cross-sectional data preclude causal inference, this finding may suggest that perceived external devaluation may exert an inward influence. This process may increase the risk of automatic negative self-activation and reflect a potential pathway from perceived stigma to internalization (Greenwald and Banaji, 2017; Epifania et al., 2022).

Several limitations should be acknowledged. The cross-sectional design prevents causal conclusions about the direction of the perceived-to-internalized pathway. In addition, the IRAP measures automatic associations under specific relational contexts; whether these associations predict behavior or longitudinal change remains to be tested. Finally, the sample was drawn from Chinese correctional settings, and the cultural specificity of the findings requires further examination.

## General discussion

Integrating explicit scale development with implicit measurement, this research delineates the dimensionality and characteristics of self-stigma among justice-involved adolescents in Chinese correctional settings. Study 1 developed and validated the Self-Stigma Scale for Adolescents in Conflict with the Law (SSS-ACL), confirming a two-dimensional structure comprising perceived public stigma and internalized self-stigma. Study 2 used the IRAP to probe implicit self-stigma, revealing widespread automatic group-negative associations (81.08%) alongside limited automatic negative self-associations (25.68%), with the two implicit indices uncorrelated. These complementary methods provide a comprehensive account of self-stigma in this population.

First, the two-dimensional structure diverges from the four-stage sequential model validated with adult offenders (Moore et al., 2016), as stereotype agreement and anticipated stigma did not emerge as separate factors. As elaborated in Study 1, this parsimonious structure likely reflects heightened peer-group salience and a more limited future time perspective during adolescence (Brown, 2004; Nurmi, 1991). Critically, the IRAP findings offer automatic-level behavioral evidence for this developmental account: a psychological buffer appears to operate between group-level and self-level stigma representations, indicating that perceived societal devaluation has not yet fully permeated the self-system. The perception-to-internalization trajectory thus remains incomplete and potentially reversible, providing a strong empirical basis for early intervention.

Second, the explicit-implicit correlation pattern supports a conditional association framework. As reported in Study 2, explicit-implicit relations varied systematically with self-relevance. In correlational terms, explicit-implicit divergence was most evident for internalized stigma, which directly threatens self-identity, whereas perceived stigma showed only marginal explicit–implicit correspondence. Notably, the small positive relation between explicit perceived stigma and implicit internalized stigma suggests a possible internalization pathway. This pattern indicates that explicit-implicit correspondence is not a fixed property but varies with self-relevance and the availability of controlled processing (Greenwald and Banaji, 2017; Greenwald and Lai, 2020; Epifania et al., 2022), extending this conditional framework into the stigma domain. At the same time, low correlations do not imply that the two systems are completely isolated; contextual sensitivity, social desirability bias, and incompatibility of measurement formats may all attenuate the consistency between explicit and implicit measures (Kurdi et al., 2023; Payne, 2026).

Third, this research provides an integrative perspective on the debate concerning whether indirect measures capture stable representations or contextually constructed evaluations (Greenwald and Banaji, 2017; Greenwald and Lai, 2020; Payne and Gawronski, 2010). Payne (2026) argues that implicit evaluations reflect stereotypical associations shared across environmental, cultural, and historical structures rather than stable individual traits. The present findings are partially consistent with this view: the robust positive D-scores for implicit perceived stigma, observed across nearly the entire sample, indicate that culturally pervasive negative group stereotypes have formed stable, chronically accessible associations, consistent with a representational account (Fazio, 1990). By contrast, the negative D-scores for implicit internalized stigma (reflecting positive self-associations) and the low detection rate for automatic negative self-linkages demonstrate that these cultural representations do not automatically transfer to self-referential processing. This pattern supports a dual-representation architecture rather than a purely representational or purely constructionist view: group-level associations, reinforced through shared cultural discourse, operate as stable long-term memory links, whereas self-referential associations are constructed under the influence of protective self-functions. Group representations and self-representations thus function as relatively independent associative networks (Greenwald and Lai, 2020). Practically, this implies that justice-involved adolescents retain considerable self-system plasticity: the same adolescent may simultaneously hold a strong automatic negative stereotype about their ingroup and a relatively intact positive self-concept. Interventions should therefore prioritize blocking internalization over merely reducing perceived public stigma, strengthening the capacity to decouple external labels from self-worth. Moreover, combining explicit screening with implicit assessment may facilitate the identification of distinct subtypes for stratified intervention.

## Limitations and future directions

Several limitations should be noted. First, the SSS-ACL was developed and validated within a Chinese collectivist cultural context, where stigma often extends beyond the individual to the family and broader kinship network. Justice-involved adolescents in China thus not only face negative evaluations of their own moral character but also subject their families to shame and social exclusion (Yang et al., 2007). This pattern contrasts with individualistic societies, where stigma is typically circumscribed to personal identity and autonomous responsibility; accordingly, the two-dimensional structure requires systematic cross-cultural validation. Second, the exclusive sampling from specialized correctional institutions limits generalizability to other justice-involved youth, and the marked gender imbalance further constrains subgroup comparisons. Third, the cross-sectional design precludes causal inferences about the perception-to-internalization trajectory. Future research should employ longitudinal designs, ecological momentary assessment, and neurophysiological measures to capture temporal dynamics, and should include more diverse and culturally varied samples to test the robustness of the perceived–internalized framework.

## Conclusion

This study developed the SSS-ACL and confirmed its sound psychometric properties, establishing perceived stigma and internalized stigma as a robust two-dimensional structure. IRAP results further revealed that while most justice-involved youth automatically perceived negative societal attitudes, these perceptions had generally not been integrated into their self-concepts, indicating that the self-system retains considerable psychological resources. A conditional dissociation was observed between explicit and implicit measures, and the small positive correlation between explicit perceived stigma and implicit internalized stigma is preliminarily consistent with the hypothesis that awareness of societal devaluation may, over time, be associated with implicit self-devaluation—a pathway requiring longitudinal testing. These findings provide theoretical and empirical foundations for destigmatization interventions in correctional education settings.

## Data Availability

The raw data supporting the conclusions of this article will be made available by the authors, without undue reservation.
