# Supplementary material for: Development of self-stigma scale for adolescents in conflict with the law and its association with implicit self-stigma
Source: Front Psychol. 2026 Sep 21;17:1767941. doi: 10.3389/fpsyg.2026.1767941 (PMC13635115; doi:10.3389/fpsyg.2026.1767941)
Supplement: Supplementary file 1 [file Supplementary_file_1.docx]

SUPPLEMENTARY MATERIALS

1. Item Analysis

**TABLE S1 Summary of Item Analysis (*n*=720)**

| Item | CR | Corrected Item-Total Correlation | Cronbach's Alpha if Item Deleted | Communality | Factor Loading | Remarks |
| --- | --- | --- | --- | --- | --- | --- |
| 1 | 9.768*** | 0.390** | 0.880 | 0.200 | 0.450 | Retained |
| 2 | 6.483*** | 0.271** | 0.881 | 0.031 | 0.175 | Deleted |
| 3 | -0.677 | -0.026 | 0.886 | 0.021 | -0.144 | Deleted |
| 4 | 5.513*** | 0.239** | 0.882 | 0.010 | 0.098 | Deleted |
| 5 | 10.753*** | 0.410** | 0.879 | 0.215 | 0.464 | Retained |
| 6 | 15.703*** | 0.548** | 0.877 | 0.401 | 0.634 | Retained |
| 7 | 15.193*** | 0.538** | 0.877 | 0.397 | 0.630 | Retained |
| 8 | 15.449*** | 0.533** | 0.877 | 0.395 | 0.629 | Retained |
| 9 | -3.717*** | -0.170** | 0.888 | 0.025 | -0.157 | Deleted |
| 10 | 17.185*** | 0.558** | 0.877 | 0.375 | 0.612 | Retained |
| 11 | 13.199*** | 0.504** | 0.878 | 0.347 | 0.589 | Retained |
| 12 | 9.905*** | 0.419** | 0.879 | 0.243 | 0.493 | Retained |
| 13 | 16.046*** | 0.540** | 0.877 | 0.345 | 0.588 | Retained |
| 14 | 10.104*** | 0.389** | 0.880 | 0.062 | 0.249 | Deleted |
| 15 | 11.512*** | 0.419** | 0.879 | 0.084 | 0.291 | Deleted |
| 16 | 13.173*** | 0.522** | 0.877 | 0.314 | 0.561 | Retained |
| 17 | 9.246*** | 0.365** | 0.880 | 0.03 | 0.175 | Deleted |
| 18 | 13.189*** | 0.488** | 0.878 | 0.327 | 0.572 | Retained |
| 19 | 7.897*** | 0.304** | 0.881 | 0.081 | 0.285 | Deleted |
| 20 | 8.934*** | 0.358** | 0.880 | 0.094 | 0.307 | Deleted |
| 21 | 14.194*** | 0.499** | 0.878 | 0.289 | 0.538 | Retained |
| 22 | 10.832*** | 0.406** | 0.879 | 0.059 | 0.242 | Deleted |
| 23 | 15.257*** | 0.564** | 0.876 | 0.330 | 0.574 | Retained |
| 24 | 4.729*** | 0.216** | 0.882 | 0.001 | 0.031 | Deleted |
| 25 | 11.807*** | 0.465** | 0.878 | 0.277 | 0.526 | Retained |
| 26 | 11.173*** | 0.468** | 0.878 | 0.216 | 0.465 | Retained |
| 27 | 8.798*** | 0.360** | 0.880 | 0.031 | 0.176 | Deleted |
| 28 | 15.852*** | 0.529** | 0.877 | 0.340 | 0.583 | Retained |
| 29 | 16.050*** | 0.543** | 0.877 | 0.332 | 0.576 | Retained |
| 30 | 15.972*** | 0.561** | 0.877 | 0.407 | 0.638 | Retained |
| 31 | 14.983*** | 0.552** | 0.877 | 0.385 | 0.620 | Retained |
| 32 | 7.914*** | 0.319** | 0.881 | 0.028 | 0.166 | Deleted |
| 33 | 16.336*** | 0.577** | 0.876 | 0.402 | 0.634 | Retained |
| 34 | 12.545*** | 0.464** | 0.878 | 0.276 | 0.526 | Retained |
| 35 | 6.594*** | 0.295** | 0.881 | 0.015 | 0.121 | Deleted |
| 36 | 17.954*** | 0.583** | 0.876 | 0.397 | 0.63 | Retained |
| 37 | 5.719*** | 0.194** | 0.883 | 0.045 | 0.213 | Deleted |
| 38 | 8.120*** | 0.372** | 0.880 | 0.113 | 0.337 | Deleted |
| 39 | 6.722*** | 0.301** | 0.881 | 0.073 | 0.270 | Deleted |
| 40 | 11.625*** | 0.490** | 0.878 | 0.273 | 0.523 | Retained |
| 41 | 6.675*** | 0.293** | 0.881 | 0.016 | 0.128 | Deleted |
| 42 | 8.347*** | 0.350** | 0.880 | 0.033 | 0.182 | Deleted |
| 43 | 8.535*** | 0.326** | 0.881 | 0.090 | 0.300 | Deleted |
| 44 | 10.079*** | 0.390** | 0.879 | 0.042 | 0.206 | Deleted |

Note：***p*＜0.01，****p*＜0.001.

2. Self-Stigma Scale for Adolescents in Conflict with the Law, SSS-ACL

**TABLE S2**

| Item | Strongly disagree | Disagree | Somewhat disagree | Somewhat agree | Agree | Strongly agree |
| --- | --- | --- | --- | --- | --- | --- |
| 1 Most people think that young people like me who have been in trouble with the law are dangerous. | 1 | 2 | 3 | 4 | 5 | 6 |
| 2 Most people are unwilling to work with someone who was once in trouble with the law, like me. | 1 | 2 | 3 | 4 | 5 | 6 |
| 3 Most people feel disgusted with young people like me who have been in trouble with the law. | 1 | 2 | 3 | 4 | 5 | 6 |
| 4 Most people think young people like me who have been in trouble with the law are untrustworthy and unreliable. | 1 | 2 | 3 | 4 | 5 | 6 |
| 5 Most people look down on young people like me who have been in trouble with the law. | 1 | 2 | 3 | 4 | 5 | 6 |
| 6 Most people do not value the suggestions or opinions of young people like me who have been in trouble with the law. | 1 | 2 | 3 | 4 | 5 | 6 |
| 7 Most people think young people like me who have been in trouble with the law are incapable of repenting. | 1 | 2 | 3 | 4 | 5 | 6 |
| 8 I worry or fear that others will find out about my offense history and experiences. | 1 | 2 | 3 | 4 | 5 | 6 |
| 9 Because of my offense history, I avoid contacting former classmates or relatives. | 1 | 2 | 3 | 4 | 5 | 6 |
| 10 I hate myself because of my past offenses. | 1 | 2 | 3 | 4 | 5 | 6 |
| 11 Even if I sincerely repent, people will not truly trust me. | 1 | 2 | 3 | 4 | 5 | 6 |
| 12 I feel that I am a danger to society or to others. | 1 | 2 | 3 | 4 | 5 | 6 |
| 13 I feel inferior because of my offense history. | 1 | 2 | 3 | 4 | 5 | 6 |
| 14 I feel that my past offenses have cost me my social standing. | 1 | 2 | 3 | 4 | 5 | 6 |
| 15 I expect that I will try my best to hide my offense history and identity. | 1 | 2 | 3 | 4 | 5 | 6 |
| 16 I feel that I will become lonelier in the future because of social exclusion. | 1 | 2 | 3 | 4 | 5 | 6 |

3. Block Sequence in IRAP

**TABLE S3 IRAP Testing Procedure for Perceived and Internalized Stigma**

| Initial Consistent Group | | Initial Inconsistent Group | |
| --- | --- | --- | --- |
| Block1 | Practice (Consistent) | Block1 | Practice (Inconsistent) |
| Block2 | Practice (Inconsistent) | Block2 | Practice (Consistent) |
| Block3 | Test (Consistent) | Block3 | Test (Inconsistent) |
| Block4 | Test (Inconsistent) | Block4 | Test (Consistent) |
| Block5 | Test (Consistent) | Block5 | Test (Inconsistent) |
| Block6 | Test (Inconsistent) | Block6 | Test (Consistent) |
